# A dual-mode dual-stator hybrid rotor synchronous and vernier machine for variable speed applications

**DOI:** 10.1038/s41598-026-40821-y

**Published:** 2026-02-24

**Authors:** Sajad Ali, Qasim Ali, Hafiz Mudassir Munir, Hasan A. Zidan

**Affiliations:** 1 Department of Electrical Engineering, Sukkur IBA University, Sukkur, Sindh 65200 Pakistan; 2 Department of Electrical and computer Engineering, College of Engineering and Information Technology, Ajman University, Ajman, 346 United Arab Emirates

**Keywords:** Vernier machine, Synchronous machine hybrid rotor, Brushless operation, Finite element analysis (FEA), Dual mode operation, Energy science and technology, Engineering, Physics

## Abstract

This paper presents the design and electromagnetic performance evaluation of a Dual-Mode Dual-Stator Hybrid-Rotor Synchronous and Vernier Machine (DMDS-HRSVM) developed for automatic washing-machine applications. The washing machine operates in two distinct cycles: a low-speed, high-torque wash cycle and a high-speed, energy-efficient spin-dry cycle. The proposed configuration combines two stators with a single hybrid rotor, allowing the machine to operate in both brushless vernier and synchronous modes within one compact structure Machines outer stator is made of 24 slots, each with either a 4 pole main winding 2 pole auxiliary winding, which combine together to create fundamental and subharmonic magnetomotive force (MMF) components in the air gap. These elements cause voltages in a 2pole rotor excitation winding by the airgap the induced AC is rectified by a rotating rectifier to the 44pole field winding which allows the rotor to run brushless. The inner stator features a 4-pole layout that interacts with permanent magnets on the rotor’s inner surface to realize synchronous operation. Finite-element analysis (FEA) confirms strong electromagnetic performance, yielding a torque of 20 Nm at 530 rpm in the vernier mode and 5.9 Nm at 1200 rpm in the synchronous mode, with corresponding efficiencies of 77% and 94.7%. These results demonstrate the DMDS-HRSVM as a compact, magnet-light, and energy-efficient alternative to existing BL-WRVM and DMDS-WRSM designs for variable-speed domestic drives.

## Introduction

The demand for energy-efficient electric drives has increased significantly during recent years for domestic applications. Variable speed applications including washing machines currently stand as an important domestic application because they need high efficiency, low noise, and high starting torque. The market uses conventional electric motors for these appliances. Researchers investigate new motor drive topologies because conventional machines struggle with several inherent problems including high cost, low compactness, and vibration issues^1^.

PMSMs maintain their status as high-efficiency and high-power-density machines. The high cost of rare-earth materials, potential for demagnetization, and environmental concerns have led the investigation into methods that reduce or eliminate the need for permanent magnets (PMs)^2,3^. The rotor flux control features of WRSMs become possible through their field windings instead of permanent magnets^4^. The traditional configuration of WRSMs has maintenance issues with brushes and slip rings, causing mechanical wear and sparking^5^.

The development of BL-WRSMs eliminates the need for brushes and slip rings^6,7^. However, these topologies often show poor starting torque and pronounced torque ripple. The recent advancements in hybrid or dual-mode machines make use of the advantages of wound rotor and PMs, yet the current systems do not incorporate efficient hybrid excitation and a smooth transition between systems^4,8^. Motor design must demonstrate excellent capabilities for varying speed applications. Should adjust its performance according to operational speeds such as washing cycles and spin dry cycles.

In^9^ synchronous and vernier machine integration is achieved using pole changing machine operates in a vernier mode at low speeds and a wound-field synchronous mode at high speeds to utilize the merits of both a vernier machine and a wound-field synchronous machine. The authors utilize two sets of windings on the rotor (or reconfigurable windings) to dynamically increase or decrease the pole numbers for Vernier and Synchronous operations within a single-stator/single-rotor structure. This approach often introduces significant control complexity.

The vernier motor is a special synchronous motor that is known for generating high torque at low speeds. Through magnetic gearing effects. The special design of this motor enables better torque density. However, conventional vernier machines often suffer from a poor power factor and also has limited efficiency at high speed^10^. This makes a vernier motor an ideal candidate for applications that need low speed but high torque, for example washing machines for washing cycles.

Washing machines serve as fundamental appliances in domestic applications. In whole world the washing machines market has strong demand and growth. Only in 2025, worldwide revenue generated by washing machines is expected to reach US$70 billion. This projected annual growth rate (CAGR 2025–2030) of 3.85%. Main contributor of this market is China generating US$18 billion revenue in 2025 alone. On a per-household basis, the market will generate US$34.05, with average ownership projected at 0.07 units per household. At the end of 2030, global unit volume is forecasted to reach 162.5 million. This is driven by an anticipated 2.2% volume growth in 2026^11^. Despite this increase in popularity of smart appliances. Traditional machines remain a main player in developing markets like in south Asia. For their affordability and user-friendly design. These trends reflect both the long-term relevance and growing technological requirement of washing machines in a wide range of consumer segments.

**Fig. 1:**
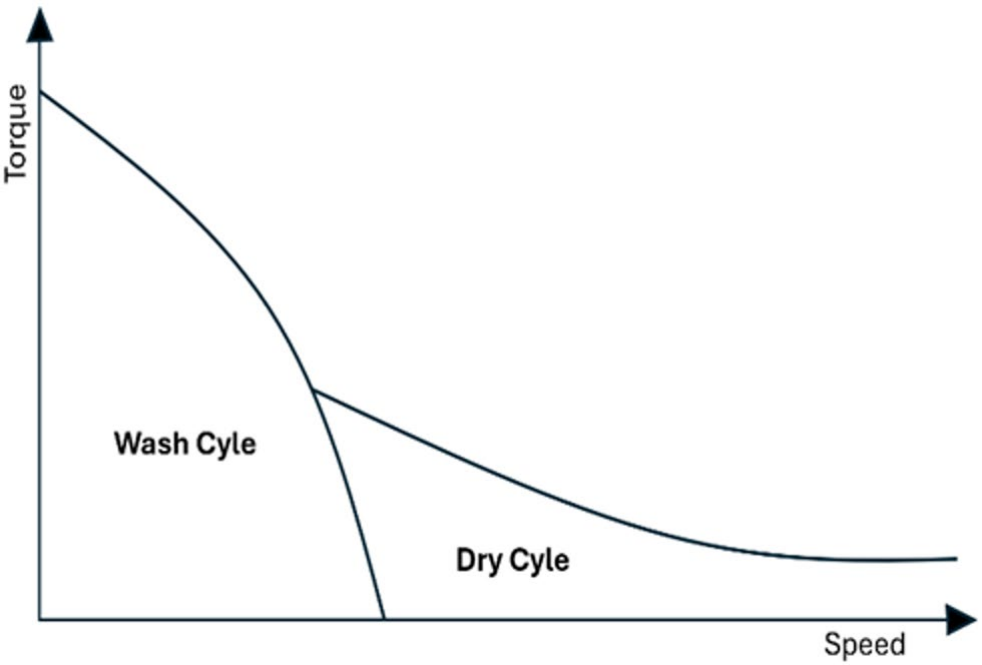
Torque-speed characteristics of washing machine motor^1^.

Washing machines function through two primary operations: the wash cycle and the dehydration (spin-dry) cycle. The wash cycle necessitates low speeds with high torque, whereas the dehydration cycle requires high speeds with low torque. Permanent Magnet Synchronous Machines (PMSM) are characterized by high efficiency at high speeds, making them particularly suitable for dehydration operations, though they are less efficient at lower speeds^12–14^. In contrast, Vernier Machines (VM) offer high efficiency at low speeds, rendering them ideal for washing operations. Notably, VMs utilize a magnetic gearing effect that yields high torque density and efficiency in the low-speed range^15–18^. Given these distinct advantages, extensive research has been conducted to propose various topologies. As shown in Fig. 1, the torque-speed characteristics highlight the dual requirement: high torque is needed at low speeds (wash cycle), while efficient high-power, low-torque operation is required at high speeds (spin-dry cycle).

Currently, induction motors (IMs) and PMSMs are used in washing machine applications. IMs have poor efficiency and torque density, and PMSMs have better efficiency but their price remains high because they rely on rare-earth magnets^3^. Hence, a DMDS-HRSVM (A Dual-Mode Dual-Stator Hybrid Rotor Synchronous and Vernier Machine) becomes a potential alternate that can provide high torque, high efficiency, and magnet-light operation.

This paper introduces a DMDS-HRSVM design for wide-range variable-speed operations. A new DMDS-HRSVM combines the advantages of hybrid excitation, brushless operation, and vernier motor principles. This leads to better performance characteristics by operating in synchronous mode for high-speed applications and vernier mode for low speed and high torque operation.

The FEA-based simulations are performed to evaluate the electromagnetic performance of the proposed DMDS-HRSVM. The rest of this research paper is structured as follows: Section II provides different brushless topologies; literature review. Section III presents the proposed model designs and proposed brushless topology along with its working principle. Section IV provides the performance analysis of the proposed machines based on 2D-FEA; Section V presents skew analysis along with comparative evaluations. Finally, Section VI provides the conclusion of this research.

## Literature review

This section discusses the development of brushless excitation topologies for conventional machines that utilize brushes and sliprings, i.e., synchronous and vernier motors. The main driving force behind the development of these brushless topologies is the maintenance problems due to the brushes and sliprings used in conventional synchronous machines for rotor excitation.

In brushless wound rotor synchronous machines (BL-WRSMs), the rotor is excited directly from within the machine without using brushes and sliprings. Besides solving the maintenance issues of conventional WRSMs, these machines achieve better excitation control capabilities. Combining improved brushless designs and optimized winding patterns has made these machines more efficient and well-suited for variable speed applications.

Various brushless excitation methods have been explored, often categorized by the harmonic component of the stator magnetomotive force (MMF) used to induce current in the rotor. Several approaches utilize higher-order harmonics, typically the 3rd or above, to induce voltage in a dedicated rotor harmonic winding^1,4,7^. Some Authors used techniques which involve inserting harmonic currents in stator windings using an specialized inverter (e.g., 3rd harmonic or 6th harmonic) with open winding stator designs, or additional switching elements like thyristors^5,7,19^ one approach is the use of anti-parallel thyristors that are switched close to current zero crossings, hence activating zero-sequence currents with a rich content of third-harmonic for the purpose of rotor excitation^7^. Another employs a second inverter to inject a single-phase 6th harmonic current at the neutral point of the main stator winding^19^. While enabling brushless operation, these higher harmonic methods can introduce control complexity and potentially increase core losses^6^.

**Fig. 2:**
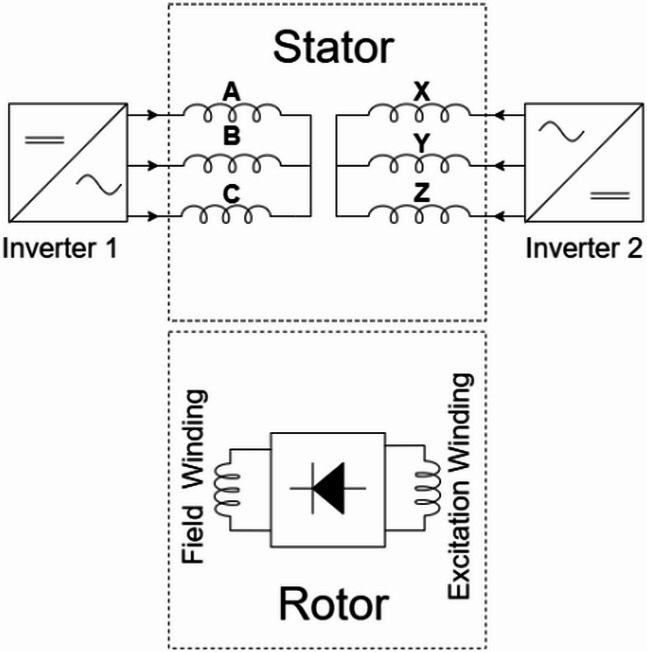
3-phase dual inverter brushless topology.

An alternative strategy employs sub-harmonic components of the stator MMF, usually at half the fundamental frequency, potentially reducing harmonic losses compared to higher-harmonic techniques^3,6,11^. A foundational sub-harmonic topology uses two inverters supplying different current magnitudes to distinguish halves of the stator winding as in Fig. 2. This current difference generates the sub-harmonic MMF needed to induce voltage in a rotor excitation winding (often 2-pole for a 4-pole fundamental field), which feeds the main field winding via a rectifier^21^. However, this spatial asymmetry in excitation can cause unbalanced radial forces (URF) on the rotor^20^.

**Fig. 3:**
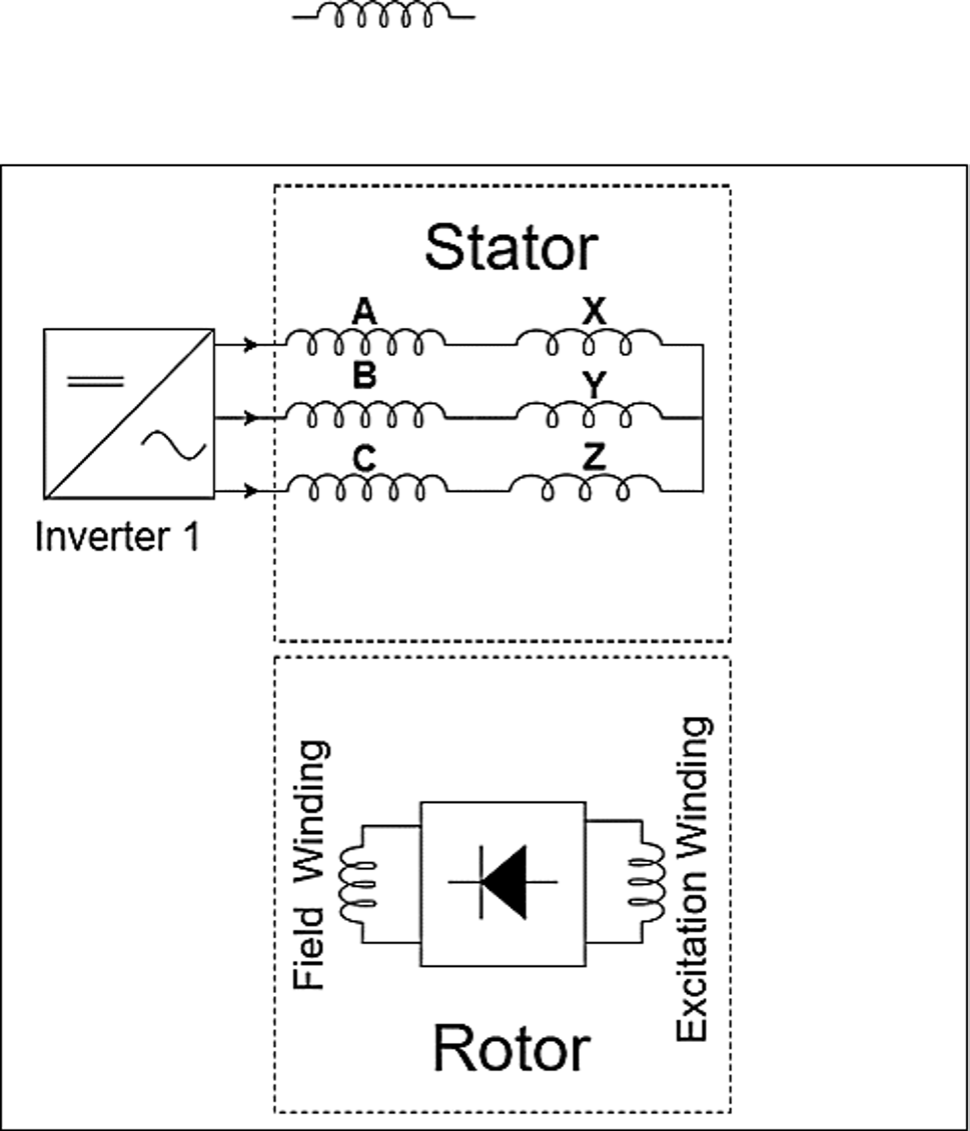
3-phase single inverter with different number of windings turns brushless topology.

**Fig. 4:**
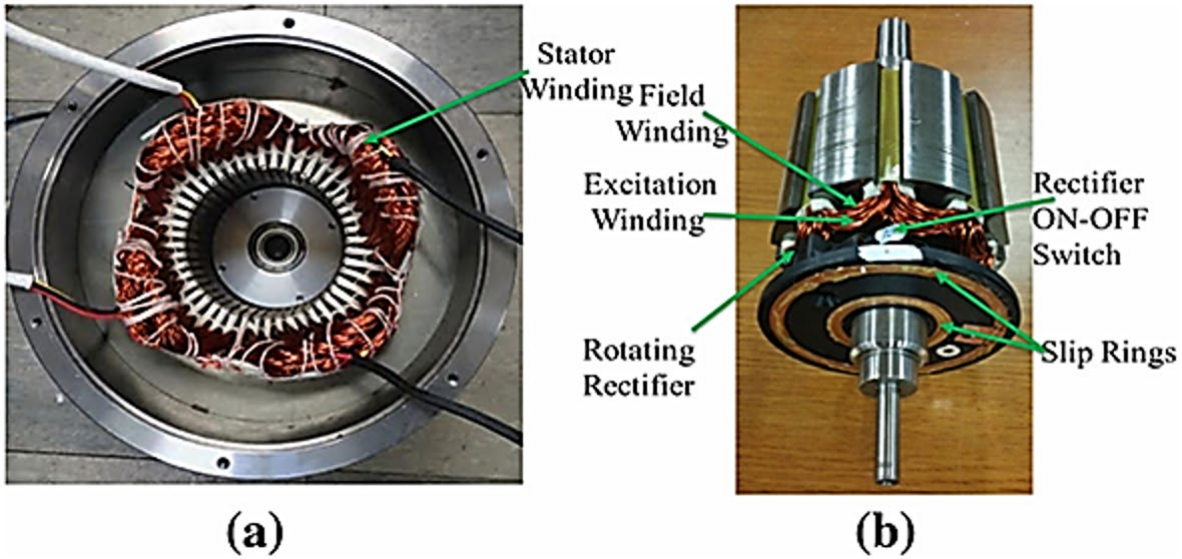
Prototype of BL-WRSM (**a**) stator and (**b**) rotor^20^.

Designing an 8-pole configuration with a more symmetric distribution of the different current windings was proposed to mitigate this URF issue^20,22^. To reduce inverter, count and cost, single-inverter topologies were developed using series-connected stator winding sections (e.g., ABC and XYZ) with unequal turns counts to inherently generate the sub-harmonic MMF as in Fig. 3. While simplifying the power electronics, this approach can lead to underutilization of stator slots^5,23^. The prototype manufactured in^20^ is shown in Fig. 4. The rotor shows the placement of rotating rectifier on one side of the rotor.

**Fig. 5:**
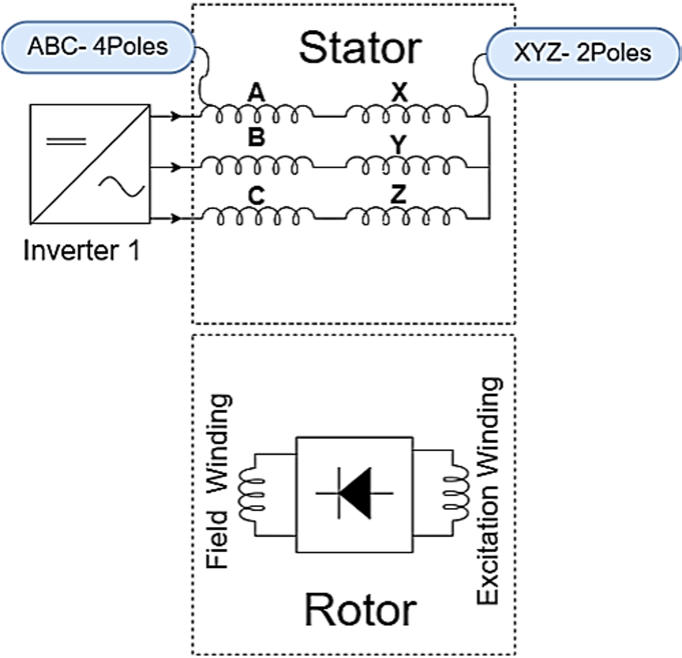
3-phase single inverter with different number of poles brushless topology.

Further refinements introduced novel multi-layer stator windings fed by a single inverter, improving slot fill factor and torque output by distributing different winding sets (e.g., 8-pole ABC and 2-pole XYZ) across layers within the slots^10,24^. Other configurations use separate main and additional stator windings with differing pole numbers (e.g., 4-pole main, 2-pole additional) fed by three-phase single-phase inverters is introduced in^1^ as shown Fig. 5. distributing these separate windings demonstrated reduced URF compared to spatially dividing a single winding.

In^25^ introduce a 36-slot stator with a 48-pole double-layer concentrated winding. A key aspect of this topology is that this specific pole-slot combination inherently generates a significant sub-harmonic component (order 0.5) in the stator magnetomotive force (MMF) alongside the fundamental component, without requiring dual inverters or special winding connections like unequal turns. This inherent sub-harmonic is then used for brushless excitation; it induces an electromotive force (EMF) in a dedicated 24-pole excitation winding on the outer rotor.

Vernier machines, known for high torque at low speeds due to magnetic gearing, offer another avenue^2,13^. While PM vernier machines (PMVMs) face cost and power factor issues^19,24^, wound rotor vernier machines (WRVMs) provide a PM-less option but require excitation^24^. Applying brushless techniques, such as sub-harmonic^19,24,26^ and third-harmonic excitation^4,21^, creates brushless WRVMs (BL-WRVMs) suitable for direct-drive applications^4,19,21,24,26^. Topologies using single inverters with combined 3-phase main and 1-phase additional stator windings have been specifically investigated for BL-WRVMs, aiming for cost-effectiveness^10^.

Brushless topologies can remove the mechanical contact; but often face such problems as low starting torque and the inability to maintain constant torque below rated speed. These difficulties are due to the natural variation of the induced field current with speed^6,8^.

To address this, permanent magnets have been added to the rotor, creating PM-assisted hybrid machines that improve starting torque and torque density^3^. Consequent pole PM arrangements offer a way to reduce the amount of expensive PM material needed^3^. This preserves constant torque capability while reducing brush wear at high speeds^8^. Dual-inverter control in such hybrid sub-harmonic machines provides enhanced field regulation capability^3^.

Another approach is the dual-mode operation concept, where the machine operates as a conventional WRSM (C-WRSM) using brushes/slip rings for external excitation in the low-speed, constant-torque region (Mode I), and switches to brushless sub-harmonic excitation (BL-WRSM) in the high-speed, constant-power region (Mode II)^8^.Switching between operating modes usually requires adjusting how the stator windings are connected, often through additional switching elements.

To achieve even higher torque density, researchers have explored combining dual-stator structures with dual-mode operation^4^. Moreover, optimizing rotor designs, such as incorporating a second harmonic winding phase in underutilized slots, has been explored to increase induced current, which potentially increasing torque ripple^20^. Torque ripple mitigation techniques like altering slot fill factors and rotor skewing are also crucial.

So far progress has been made in development of different brushless excitation schemes for WRSMs and WRVMs. Key problems are simplifying the topology, eliminating URF, improving torque characteristics (starting torque, torque ripple, average torque density), and efficiency across wide speed ranges. While hybrid PM assistance and dual-mode configurations have addressed specific operational limitations such as extending speed ranges, enhancing torque density through dual-stator structures, and improving start-up torque via hybrid excitation, the integration of synchronous and vernier principles into a single topology remains unexplored. To address this gap, this paper introduces the Dual-Mode Dual-Stator Hybrid Rotor Synchronous and Vernier Machine (DMDS-HRSVM). This proposed architecture synthesizes these advanced features to deliver a high-performance, brushless solution that minimizes reliance on permanent magnets. It is specifically designed for washing machine applications, which necessitate distinct performance profiles for high-torque operations at low speeds and low-torque operations at high speeds^27^.

## Proposed model and proposed topology

**Fig. 6:**
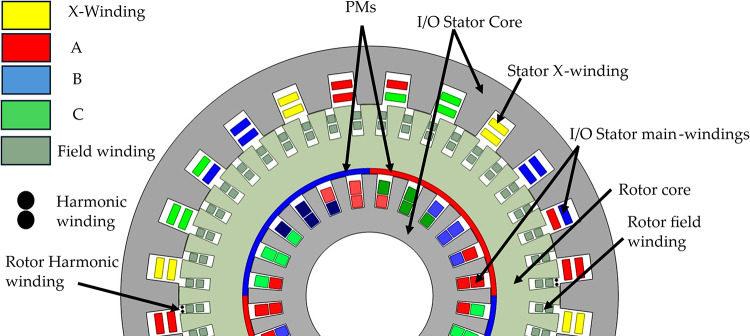
Proposed dual mode dual stator hybrid rotor synchronous and vernier machine (DMDS-HRSVM).

The proposed machine features a dual-stator, single-rotor configuration designed for dual-mode operation, integrating both synchronous and vernier principles. The machine structure comprises an inner stator, an outer stator, and a shared rotor positioned between them, as illustrated in Fig. 6.

The outer stator consists of 24 slots. Its winding arrangement is specifically designed to enable both vernier operation and brushless excitation. A 4-pole (2 pole pairs) main armature winding (ABC) occupies 18 slots supplied from three phase inverter and is primarily responsible for generating the fundamental magnetomotive force (MMF) for vernier torque production. The remaining 6 slots house a 2-pole (1 pole pair) auxiliary excitation winding (X), configured to produce a subharmonic MMF component necessary for brushless operation. For the excitation of rotor, generally 2% of input power is required, and for very large machines, it ranges from 0.17% to 2%^28^. So, the designed X winding can sufficiently excite the rotor field winding.

**Fig. 7:**
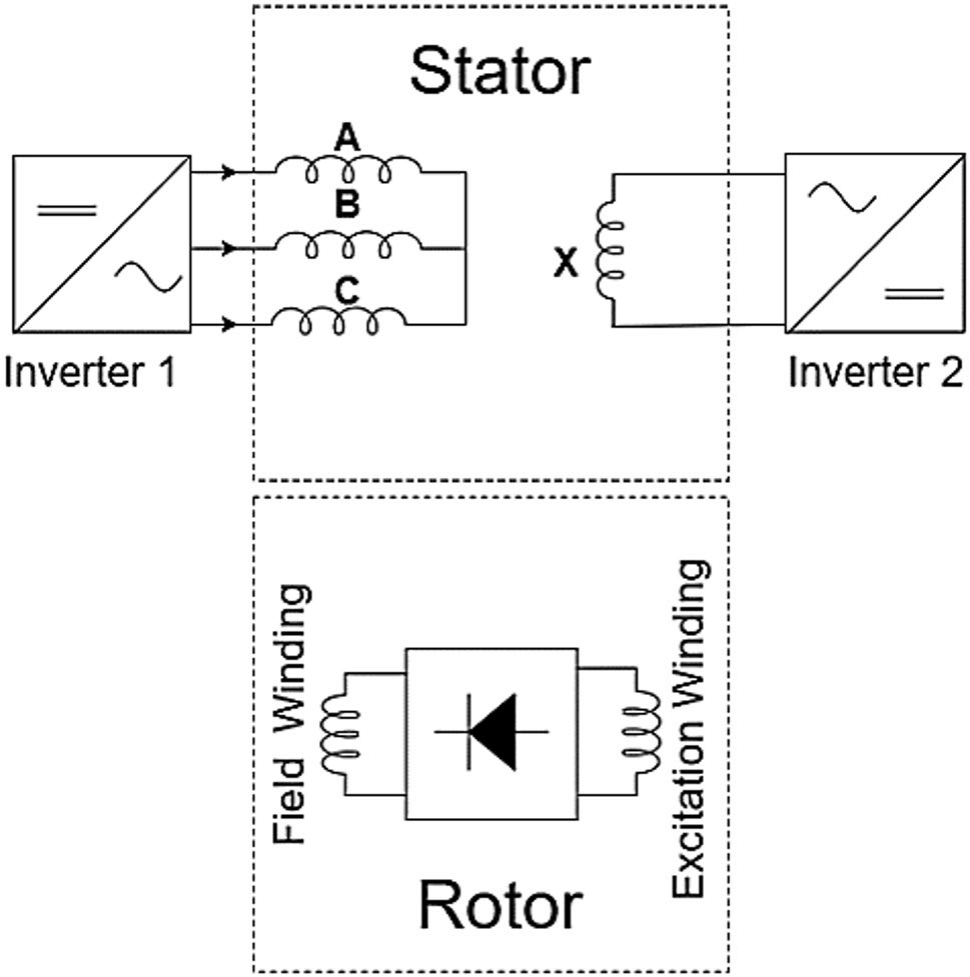
Proposed brushless topology utilizing a 3 and 1-phase inverter.

The rotor functions as a unified structure featuring distinct topologies on its interior and exterior faces. To facilitate synchronous interaction with the inner stator, the inner surface is equipped with four-pole permanent magnets. Conversely, the outer surface incorporates 44 slots, which have been enlarged relative to the reference model^1^ by leveraging the greater spatial area available at the outer periphery, and accommodates two windings for vernier operation and brushless excitation: a 44 pole (22 pole pairs) field winding and a 2 pole (1 pole pair) excitation winding. The outer stator (24 slots, 4-pole main winding) and the outer rotor (44 slots, 44-pole field winding) combination facilitates vernier motor operation. This slot/pole configuration satisfies the vernier principle, expressed as in Eq. (1).

1 $$\:{P}_{r}={P}_{s}\pm\:N$$

where Pr represents the number of rotor pole pairs (22), Ps is the number of stator slots (24), and N is the number of stator’s winding pole pairs (2 for the 4-pole main winding), confirming 22 = 24 − 2. The brushless operation of outer stator topology is adopted from reference model^1^ shown in Fig. 7, The 2-pole auxiliary winding supplied from single phase inverter on the outer stator induces voltage in the 2-pole excitation winding on the rotor via the subharmonic MMF, enabling brushless supply to the 44-pole field winding through a rotating rectifier. The key design parameters for the proposed dual-mode, dual-stator machine is detailed in Table I.

This section may be divided by subheadings. It should provide a concise and precise description of the experimental results, their interpretation, as well as the experimental conclusions that can be drawn (Table 1).

**Table 1:** Parameters of proposed model.

Parameter	Unit	Value
High Speed	rpm	1200
Low Speed	rpm	530
Inner Stator inner diameter	mm	50
Inner Stator outer diameter	mm	95.5
Outer Stator inner diameter	mm	150.5
Outer stator outer diameter	mm	196
Rotor inner diameter	mm	96
Rotor outer diameter	mm	150
Stack length	mm	100
Airgap length with inner stator	mm	0.5
Airgap length with outer stator	mm	0.5
Number of stator slots (inner and outer)	–	24
Number of rotor slots	–	44
Stator winding poles of X	–	02
Stator winding poles of ABC		04
Excitation winding poles	–	02
Field winding poles		44
Inner Stator’s No. of turns/slot	–	18
Outer Stator’s No. of turns/slot		33
Field winding’s No. of turns/slot	–	19
Excitation winding’s turns/slot	–	5

**Fig. 8:**
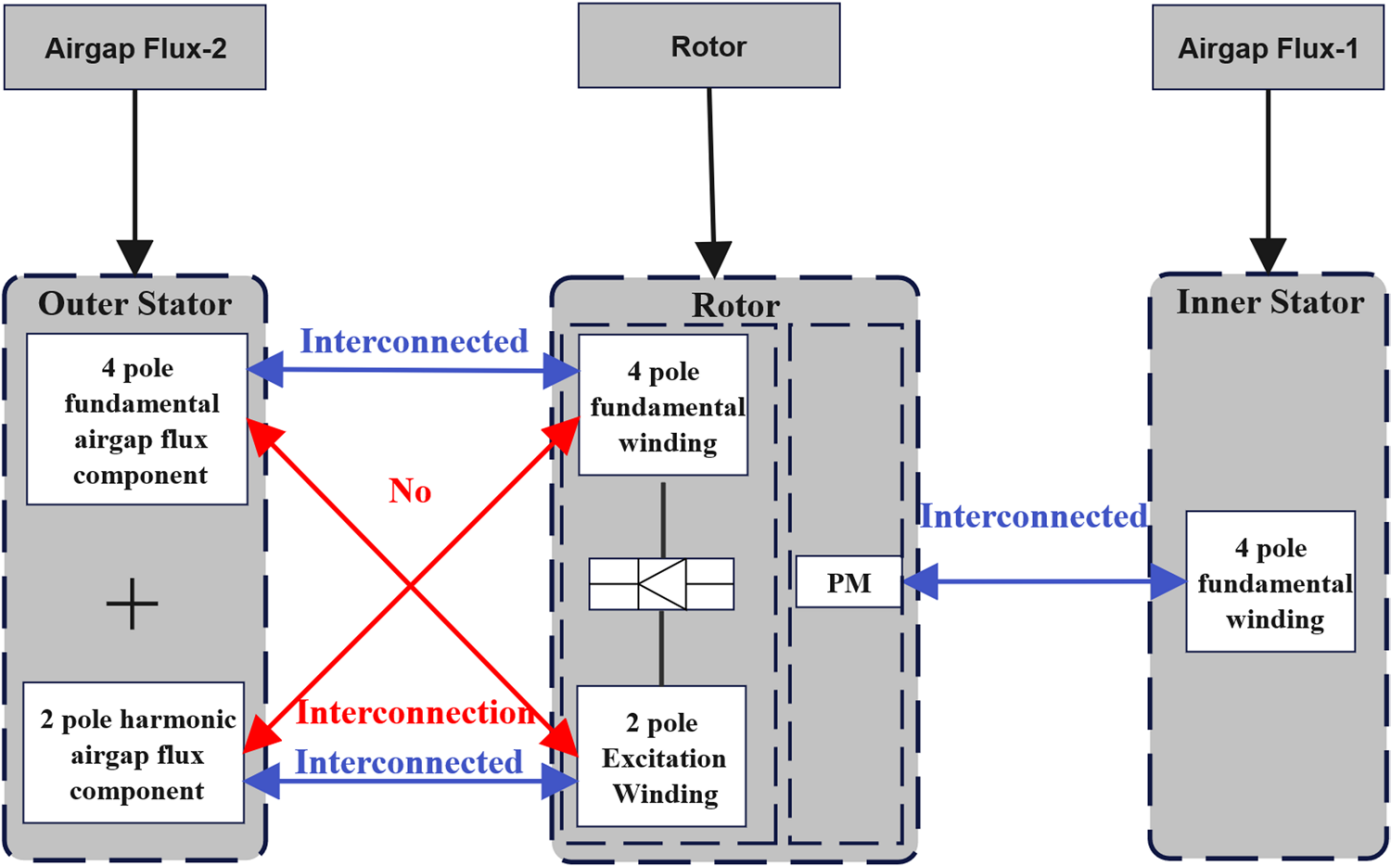
Working principle of brushless operation.

The working principle of the proposed motor is shown in Fig. 8. The outer stator is equipped with two sets of windings: a three-phase ABC winding giving the four-pole fundamental airgap flux component, and an X winding giving a two-pole subharmonic airgap flux component. These components of the fluxes are electromagnetically coupled with the field winding of 44 poles and the excitation winding of 2 poles on the rotor, respectively. The net electromagnetic interaction between the rotor and outer stator defines brushless wound-rotor operation. Further the inner stator with single three-phase ABC winding carries out electromagnetic interaction with the PMs of the rotor and produces the torque. This interaction of fluxes within both the interfaces of the air gaps allows effective torque generation and brushless excitation without mechanical brushes or slip rings in dual mode.

Since both the 2-pole excitation winding and the 44-pole field winding are located on the rotor, their interconnection is direct and brushless. The terminals of the excitation winding are connected to the AC input side of the rectifier module, and the DC output terminals of the rectifier feed the main field winding.

The rectifier module is mounted on the rotor endplate, as demonstrated in the prototype in Reference^20^, Fig. 4. To ensure mechanical stability and avoid unbalanced forces during rotation, a counterweight can be installed on the rotor’s opposite side.

There is no torque generated by the interaction between the stator X winding and the rotor excitation winding. This is because of the fact that rotor excitation winding as well as the stator X winding, both are single phase AC windings which do not produce unidirectional Torque.

**Fig. 9:**
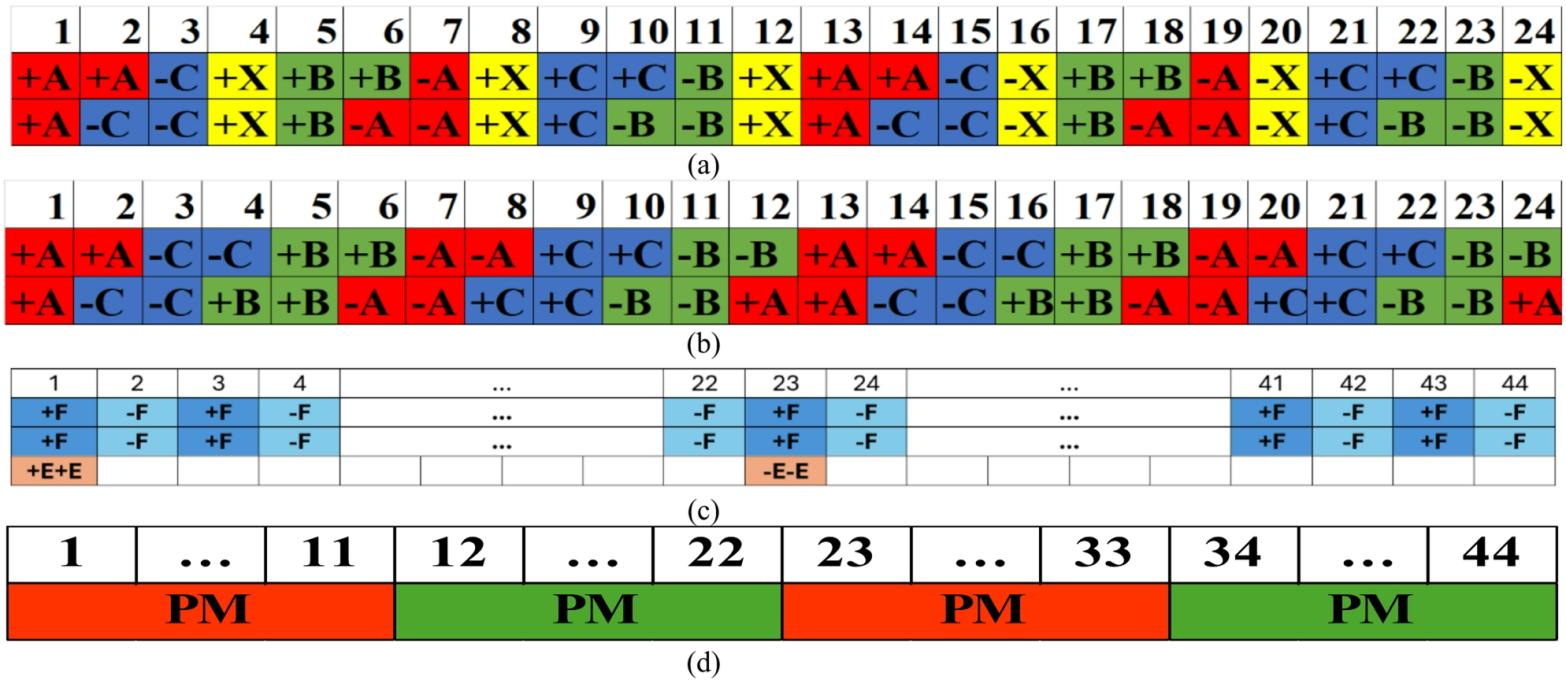
Winding layouts of stators and rotor (**a**) Outer stator (**b**) Inner stator and (**c**) Rotor field winding (**d**) Rotor PMs.

The winding configuration of the outer stator in the proposed machine is same that of the reference machine, as depicted in Fig. 9a. In contrast, the inner stator utilizes a modified layout shown in Fig. 9b, where the slots are fully occupied by the main ABC winding to maximize slot utilization. The rotor configuration is detailed in Fig. 9c,d. The rotor field winding, illustrated in Fig. 9c, features alternating polarity across 44 poles (labeled 1 through 44), alongside specific excitation winding placements (+ E+E and -E-E). Furthermore, Fig. 9d illustrates the placement of the permanent magnets (PM), arranged in alternating polarity segments spanning 11 slots each, to facilitate the hybrid excitation mechanism for inner side.

**Fig. 10:**
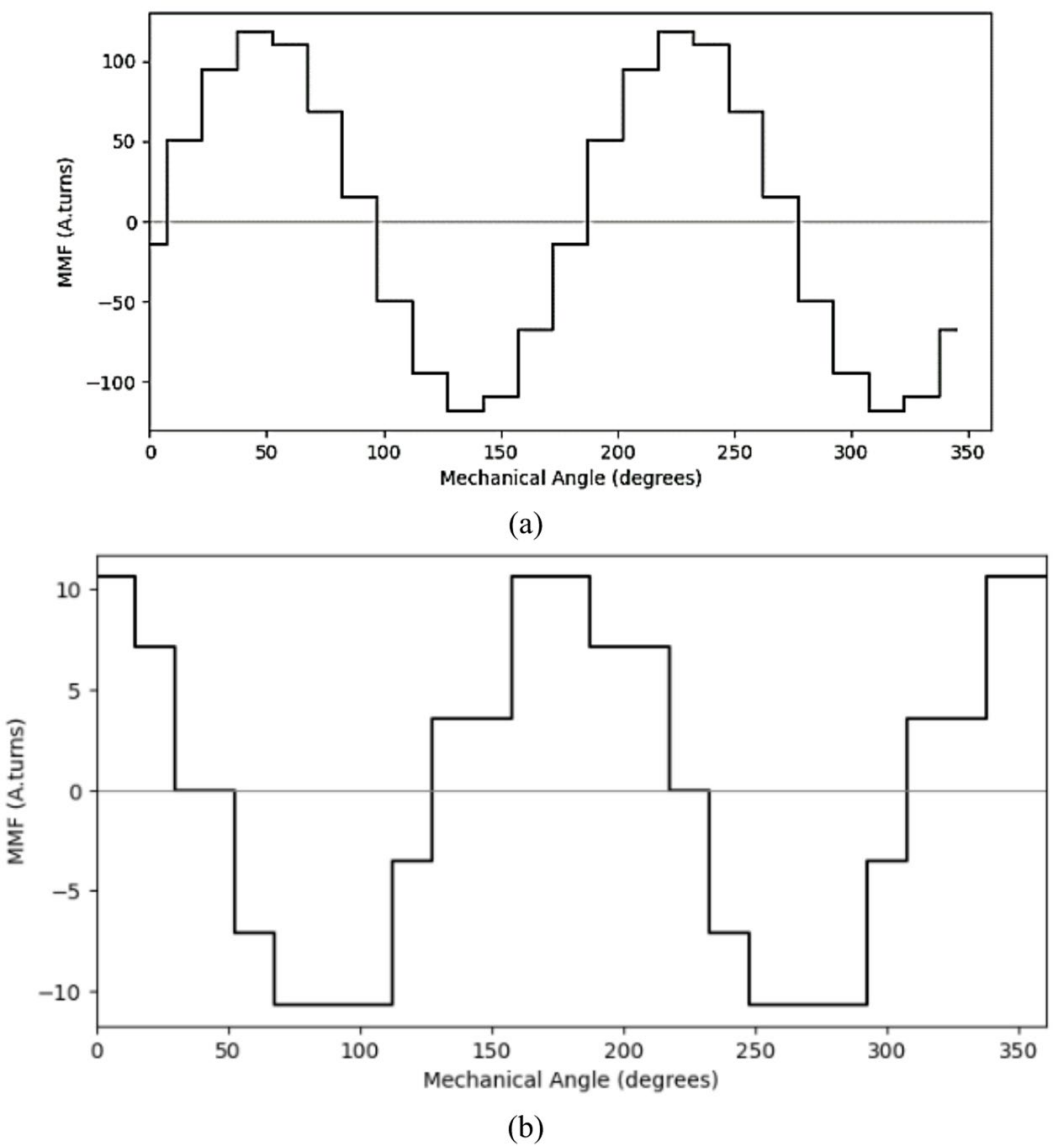
MMF plot of (**a**) inner stator (**b**) outer stator windings.

MMF plot of both inner and outer stator are shown in Fig. 10a,b respectively. The plot shows the winding function of stator windings, based on amp-turns distribution over the mechanical angle.

2 $$\:{i}_{A}={I}_{pk}\mathrm{sin}\left(\omega\:t\right)$$

3 $$\:{i}_{B}={I}_{pk}\mathrm{sin}\left(\omega\:t-\frac{2\pi\:}{3}\right)$$

4 $$\:{i}_{C}={I}_{pk}\mathrm{sin}\left(\omega\:t-\frac{4\pi\:}{3}\right)$$

5 $$\:{i}_{X}={I}_{pkx}\mathrm{sin}\left(\omega\:t\right)$$

The resulting MMF of reference and proposed machines’-based input current and winding functions are given by Eq. (6), where φ is the MMF angle, ω is the angular frequency, t is time, I_mag_ denotes the magnitude of current, N_1_ and N_2_ are number of turns in main 3 phase ABC and single phase X winding respectively, the first part of equation represents fundamental component of MMF for generation of torque and second part for subharmonic component these sub harmonics are utilized to induce current in rotors’ excitation winding for brushless operation of outer stator and rotor.

6 $$\:F\left(\phi\:,i\right)=\left[\frac{4{N}_{1}{I}_{mag}}{\pi\:}\left\{cos\left(\phi\:\right)sin\left(\omega\:t\right)+cos\left(\phi\:-\frac{2\pi\:\:}{3}\right)sin\left(\omega\:t-\frac{2\pi\:}{3}\right)+cos\left(\phi\:-\frac{4\pi\:\:}{3}\right)sin\left(\omega\:t-\frac{4\pi\:}{3}\right)\right\}+\left\{\frac{2{N}_{2}{I}_{mag}}{\pi\:}\left(cos\left(\frac{\phi\:}{2}\right)sin\left(\omega\:t\right)\right)\right\}\right]$$

In Eq. (6), the use of pole pair number of $\:\frac{1}{2}$ only represents that the X winding has half number of poles compared to the main winding (ABC winding). Now, as the X winding in the stator is 2-pole, the rotor excitation winding is also, 2 pole. The main winding produces main component of MMF which rotates with the speed of Nsf which can be calculated by Eq. (7), where frequency, f = 192 Hz and P is 44 poles. For the speed of harmonic component, Eq. (8) is used. The variable ‘h’ denotes the harmonic order and Nsh denotes the speed with which the harmonic component of MMF rotates.

7 $$\:Nsf=\frac{120*f}{P}=524\:rpm$$

8 $$\:Nsh=\frac{Nsf}{h}\:\:$$

Here h = 0.5 because we are using subharmonic therefore,

$$\:Nsh=1048\:rpm$$

Now, we see that the fundamental MMF component has the speed of 524 rpm while the speed of Sub-harmonic component is 1048 rpm. The rotor rotates at 524 rpm. Hence the sub-harmonic has 2 times the speed of rotor, which means that this component can induce the voltages on the 2-pole excitation winding of the rotor.

The speed of rotor is 524 rpm, while the speed of X winding MMF is 1048 rpm. The speed difference does not allow both components to lock together.

**Operational Strategy and Mode Switching** The proposed DMDS-HRSVM is tailored specifically for the operational cycle of automatic washing machines, which inherently separates the washing and dehydration phases with a distinct pause for water drainage. Unlike traction applications that require seamless dynamic transitioning, the washing machine cycle involves bringing the rotor to a complete standstill between the low-speed wash mode and the high-speed spin-dry mode. Consequently, the switching mechanism between the outer stator (Vernier mode) and the inner stator (Synchronous mode) is executed sequentially at zero speed. This “stop-and-switch” strategy naturally eliminates the risks of torque discontinuity and transient instability. Therefore, the proposed topology does not require the complex robust control algorithms typically necessary for dual-mode machines that must transition under load; a simple sequence control is sufficient to energize the respective stator windings based on the cycle stage.

In the high-speed (spin-dry) mode (1200 rpm), the outer vernier machine is inactive (de-energized). The machine operates purely as a Permanent Magnet Synchronous Machine (PMSM) using the inner stator and rotor magnets. The copper of the outer stator will not be used for the high-speed mode.

## Results and analysis

To evaluate the performance of the proposed machine design, finite element analysis (FEA) was conducted using Ansys Maxwell 2D simulations. The primary focus of this analysis was to characterize key performance metrics, specifically the output torque generated by the machine and the field current successfully supplied through the novel brushless excitation topology.

### No load analysis

This section discusses the no-load analysis of proposed machine conducted to evaluate the induced Back-EMF in each stator.

**Fig. 11:**
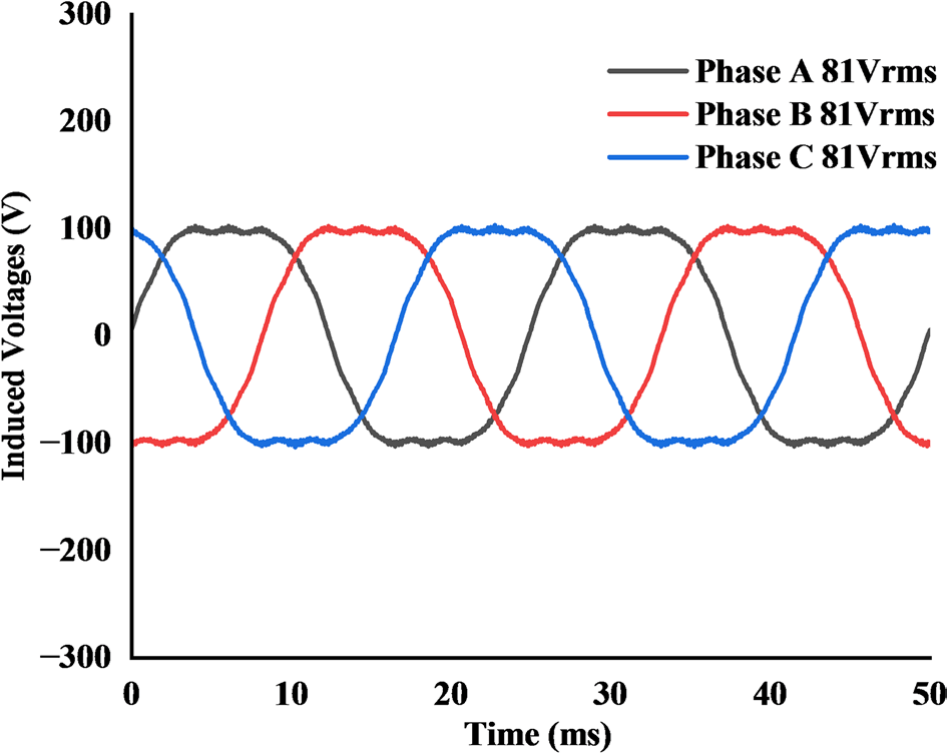
No-load induced Back-EMF in inner stator windings at 1200 rpm.

Firstly, with the inner stator synchronous operation was assessed. The rotor was driven at the high-speed value of 1200 rpm. Under this condition, the permanent magnets on the inner rotor surface induced a Back-EMF of 81 V (RMS) in the inner stator windings, as shown in Fig. 11.

**Fig. 12:**
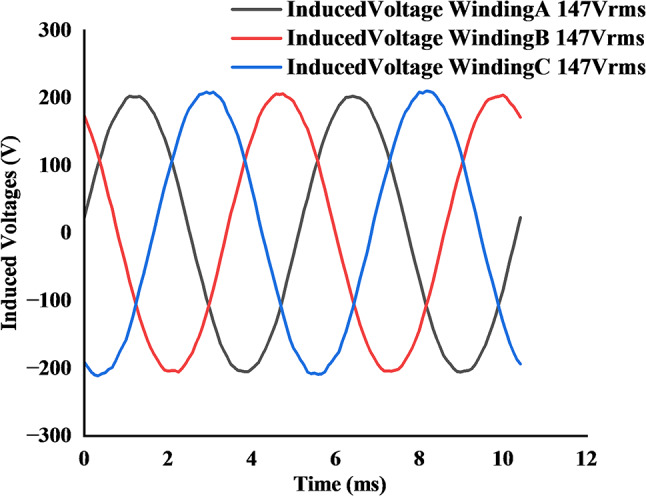
No-load induced Back-EMF in outer stator windings at 524 rpm.

Next, the no-load characteristics of the outer stator’s vernier operation were evaluated at the low speed setpoint of 524 rpm. For this test, the 44-pole outer rotor field winding was energized with 11 A DC current. This excitation produced an induced RMS voltage of 147 V in the open-circuited outer stator windings, as depicted in Fig. 12.

### Load analysis

The proposed machine’s performance was evaluated under load conditions corresponding to its two distinct operational modes: the low-speed (wash mode) and the high-speed (spin-dry mode).

**Fig. 13:**
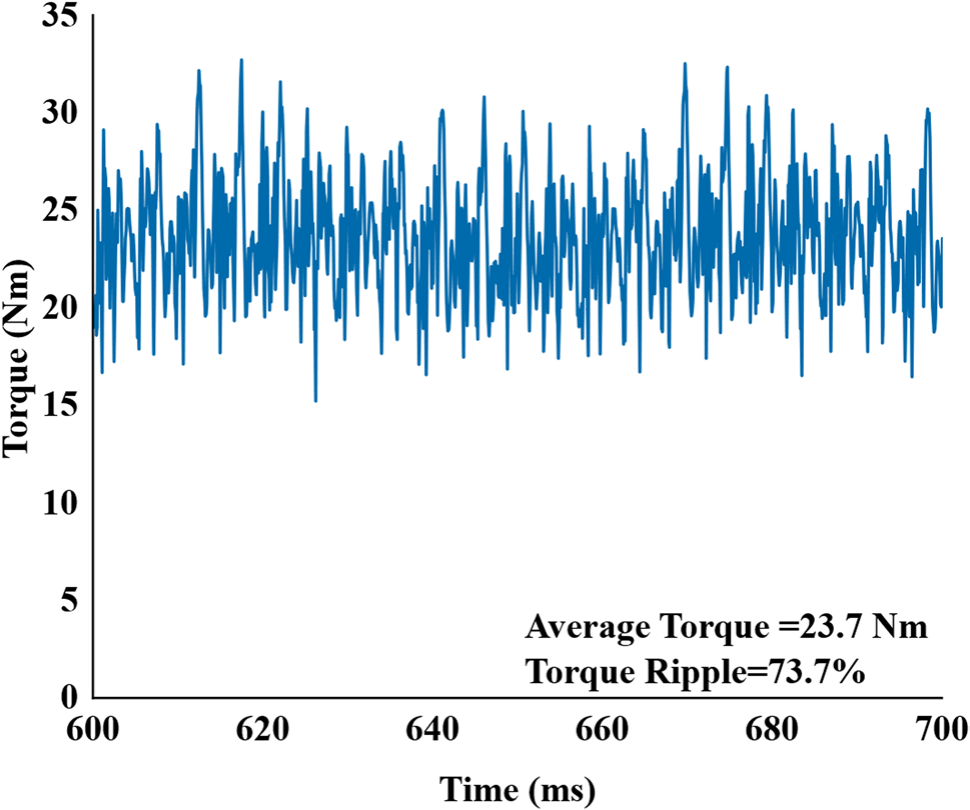
Output torque of proposed machine in low-speed wash mode of operation.

For the low-speed wash mode, the model was simulated at 530 rpm. In this mode, the outer stator’s auxiliary winding (X) was supplied with a single-phase AC current with a peak value of 1.52 A, to generate the necessary subharmonic MMF for brushless excitation and main ABC winding with 3 phase AC current with a peak value of 4.1 A to generate the fundamental MMF for torque generation. The resulting output torque characteristic is presented in Fig. 13. Under these conditions, the machine produces a high average torque of 23.7 Nm, suitable for the wash cycle, it exhibits a torque ripple of 73.7%.

**Fig. 14:**
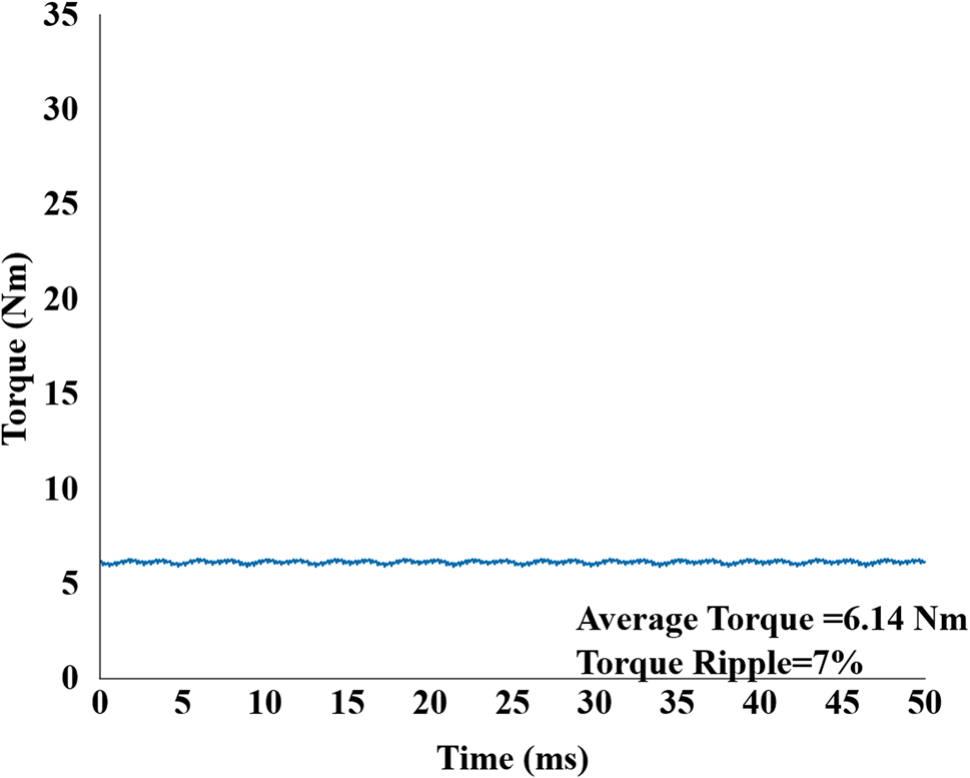
Output torque of proposed model in high-speed dry mode of operation.

For the high-speed spin-dry mode, the simulation was conducted at 1200 rpm. This mode utilizes synchronous PM operation; the inner stator winding was supplied with a current of 4.1 A peak, as shown in Fig. 14 the machine produced an average torque of 6.14 Nm with a torque ripple of 7%.

Figure 16 illustrates the currents generated by the brushless excitation system during the low-speed (wash mode) operation at 530 rpm. The harmonic current, induced in the 2-pole rotor excitation winding by the stator’s subharmonic MMF, is shown to be an AC waveform with an RMS value of 7.3 A. The induced current is then passed through the rotating rectifier, producing the DC field current required for the main 44-pole winding. The simulation shows this field current stabilizing after an initial transient, achieving an RMS value of 11.7 A. This successfully established field current interacts with the main stator field to produce the high torque required for the wash cycle.

**Fig. 15:**
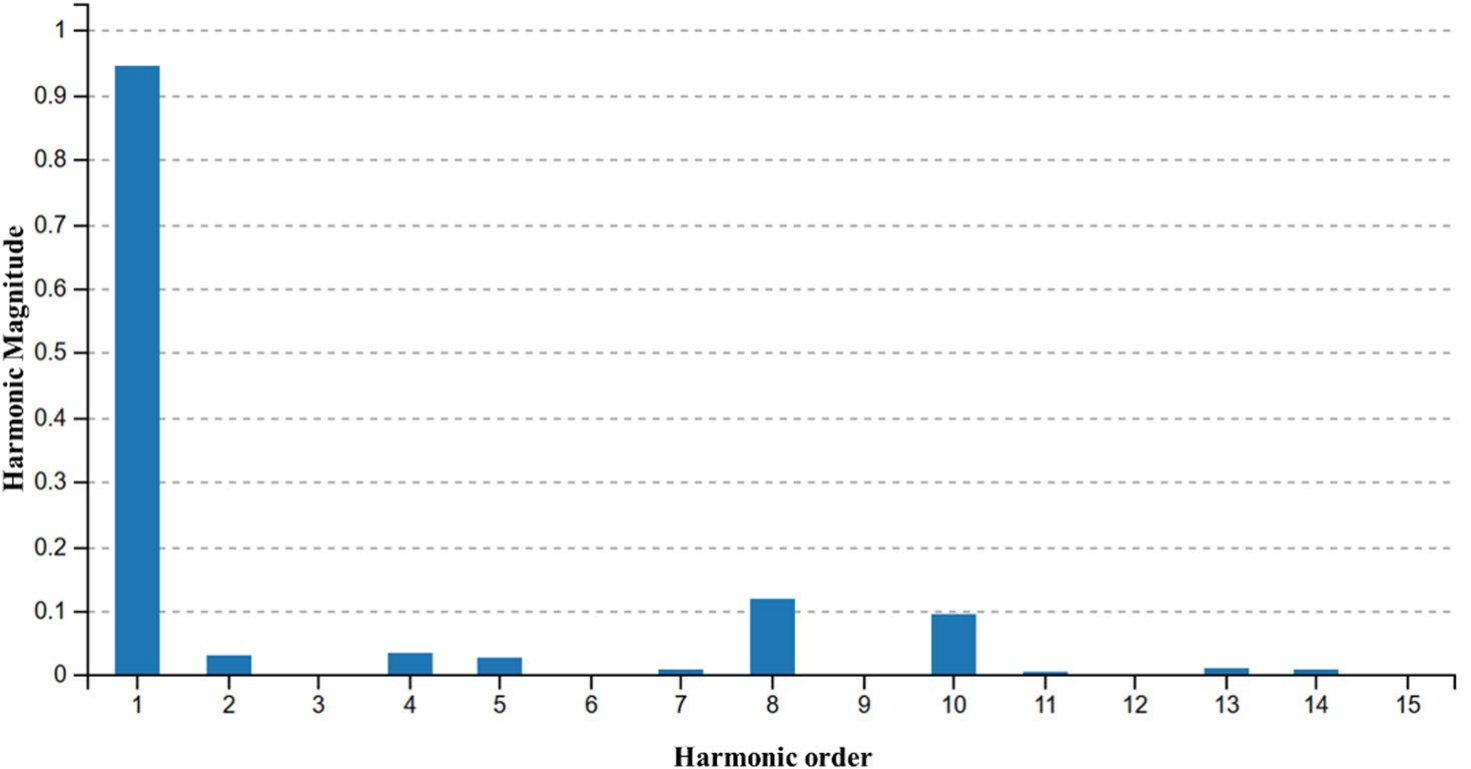
Harmonic spectrum of the stator MMF showing the fundamental and higher-order harmonics caused by the 18-slot/4-pole configuration.

**Fig. 16:**
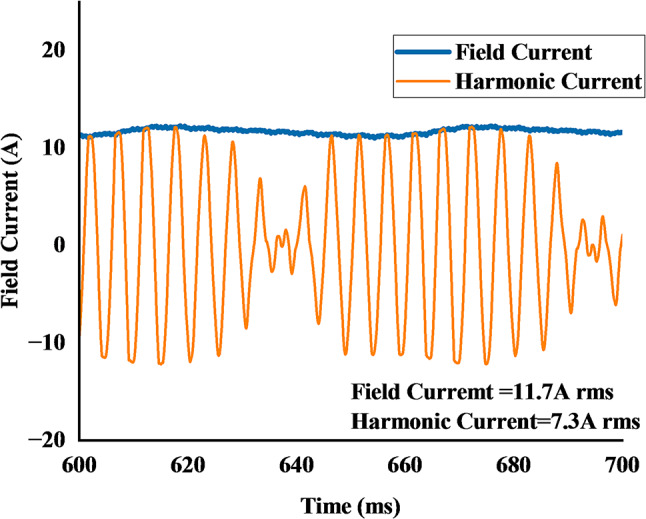
Rotor harmonic and field current of proposed model.

Further the root causes of the high torque ripple of (73.7%) identified by two primary factors:

1. **Stator MMF Harmonics (18-Slot/4-Pole)**: The main outer stator utilizes an 18-slot/4-pole fractional-slot winding configuration. While this configuration is compact, it inherently produces significant MMF space harmonics alongside the fundamental component. As illustrated in the harmonic analysis performed in Figure. 15, these localized space harmonics interact with the rotor permeance to create substantial torque pulsations.

2. **Field Current Ripple**: The brushless excitation method relies on the harmonic winding to induce voltage, which is then rectified. As shown in Figure. 16, the resulting field current is not a perfectly smooth DC but contains inherent ripple components due to the rectification process. This current ripple directly modulates the air-gap flux, further contributing to the total torque ripple.

A notable characteristic observed in the proposed DMDS-HRSVM topology is the magnitude difference between the stator excitation current and the rectified rotor field current. As evidenced in Fig. 16, an induced AC excitation current of 7.3 A (RMS) results in a DC field current of 11.7 A. This high current gain behavior is observed in recent literature on brushless wound-rotor vernier machines. For instance, Zulqarnain et al^29^.validated this characteristic, reporting that a 19.35 A (RMS) rotor harmonic current drives a 25.51 A field current in a third-harmonic excitation topology. These studies confirm that the observed current amplification in the proposed DMDS-HRSVM is a distinct and predictable advantage of the topology, allowing for high torque density.

Ali et al^23^.reported a design where a stator current of 1 A produced a field current of 10.56 A. Similarly, experimental results by Hammad et al^30^.demonstrated that a 6.34 A stator current produced a 20.55 A DC field current.

### Skew analysis

**Fig. 17:**
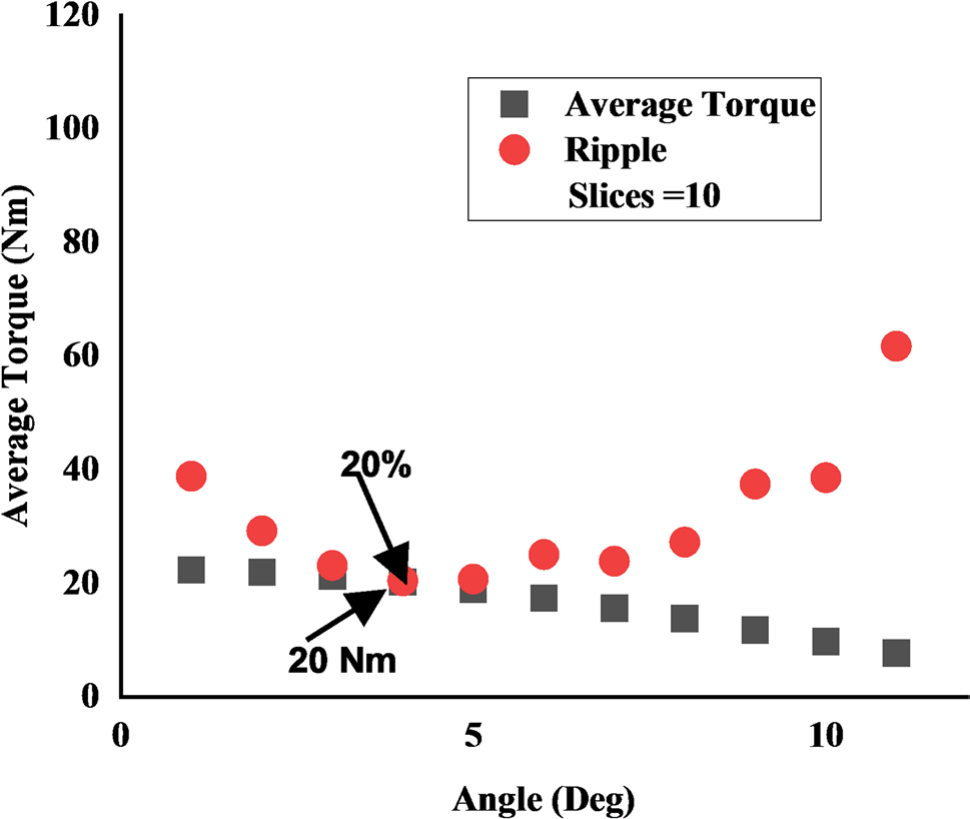
Rotor skew analysis showing the effect of skew angle on average torque and torque ripple.

For reducing the high torque ripple (73.7%) identified in the low-speed wash mode; The rotor is skewed, for this the skew angle varied from 1 to 11 degrees. The machine is operated in the conventional WRSM mode at 11 A (rms) rotor field current. Figure 17 shows the relation between average torque and torque ripple with changing skew angle. With increasing angle, the average torque decreases, however, the torque ripple decreases to a certain point before increasing again. As from the figure at 4 degrees an optimal point where torque ripple significantly reduced to 20% and produced an average torque of 20 Nm. On these parameters of a 4-degree skew angle, the final brushless model for both operating modes were evaluated.

**Fig. 18:**
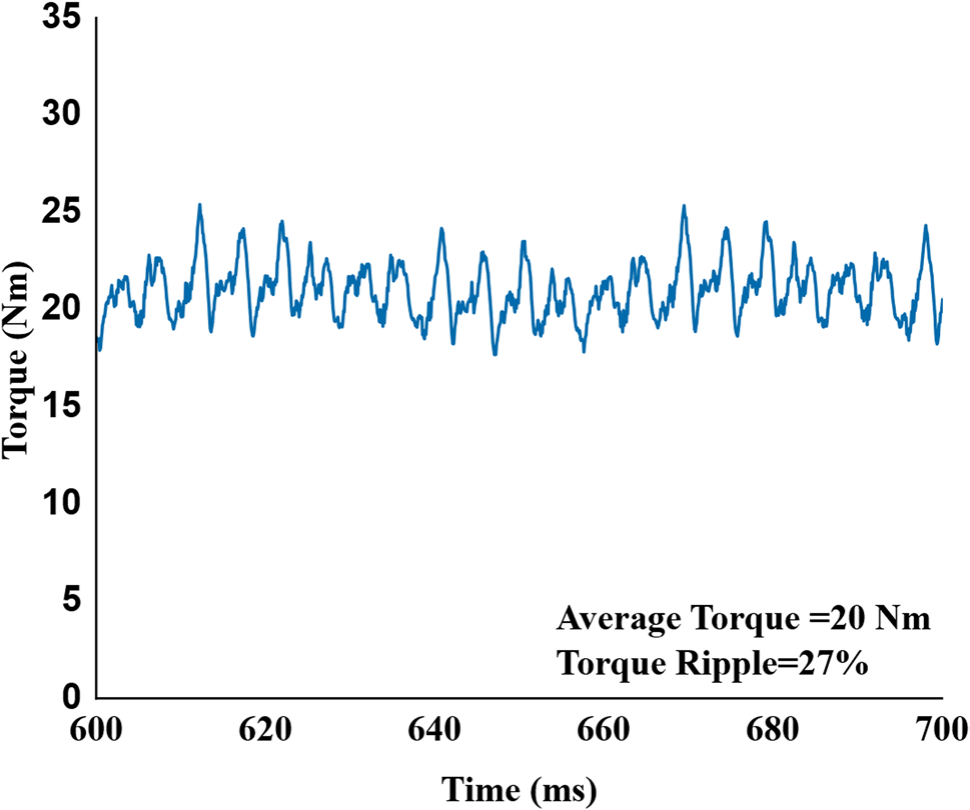
Output torque of proposed skewed model in wash mode operation with average torque 20 Nm and 27% ripple.

This analysis proved effective as shown in Fig. 18, in which the brushless wash mode torque ripple was successfully decreased to 27% and maintaining the target average output torque of 20 Nm. The same skew parameters used for the high-speed dry mode yielded a good performance. As seen in Fig. 20, this mode produced a stable average torque of 5.9 Nm with a torque ripple of 6.5%.

**Fig. 19:**
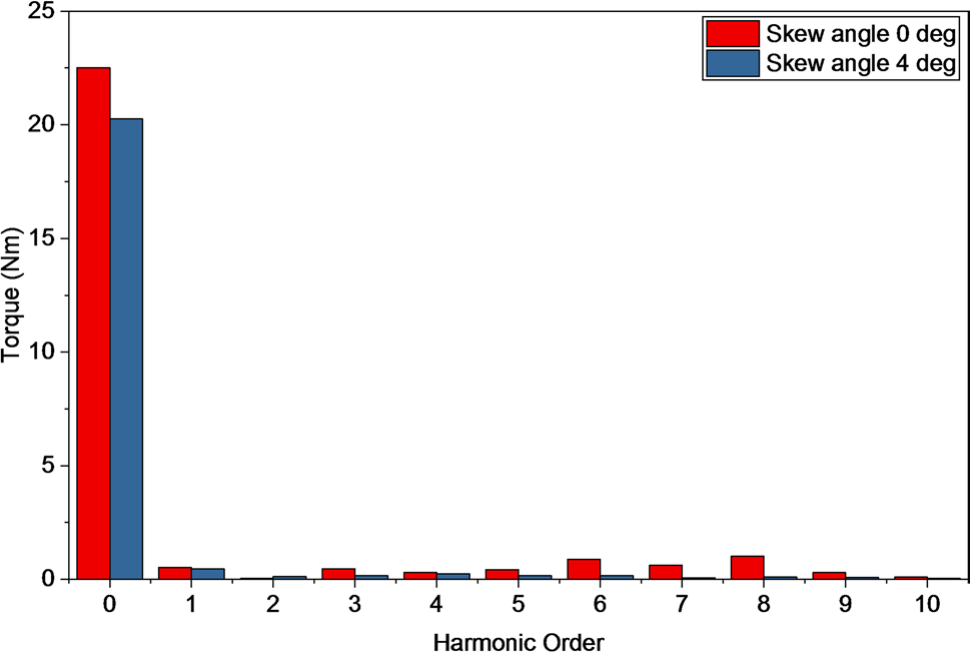
Harmonic spectrum of the output torque on skew angle of 0 deg and 4 deg.

Figure 19 shows the harmonic analysis at skew angle of zero deg and four deg. From this its clear that harmonics like 8th and 10th order, are supressed by 4 deg skew.

**Fig. 20:**
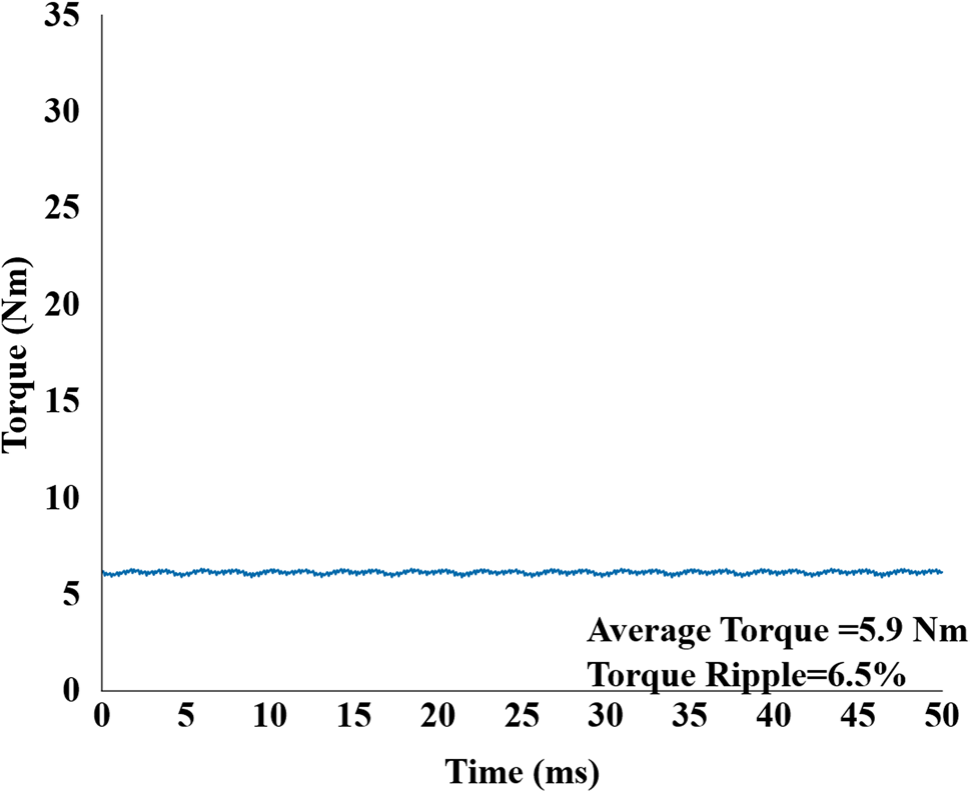
Output torque of proposed skewed model in dry mode operation with average torque 5.9 Nm and 6.5% ripple.

**Fig. 21:**
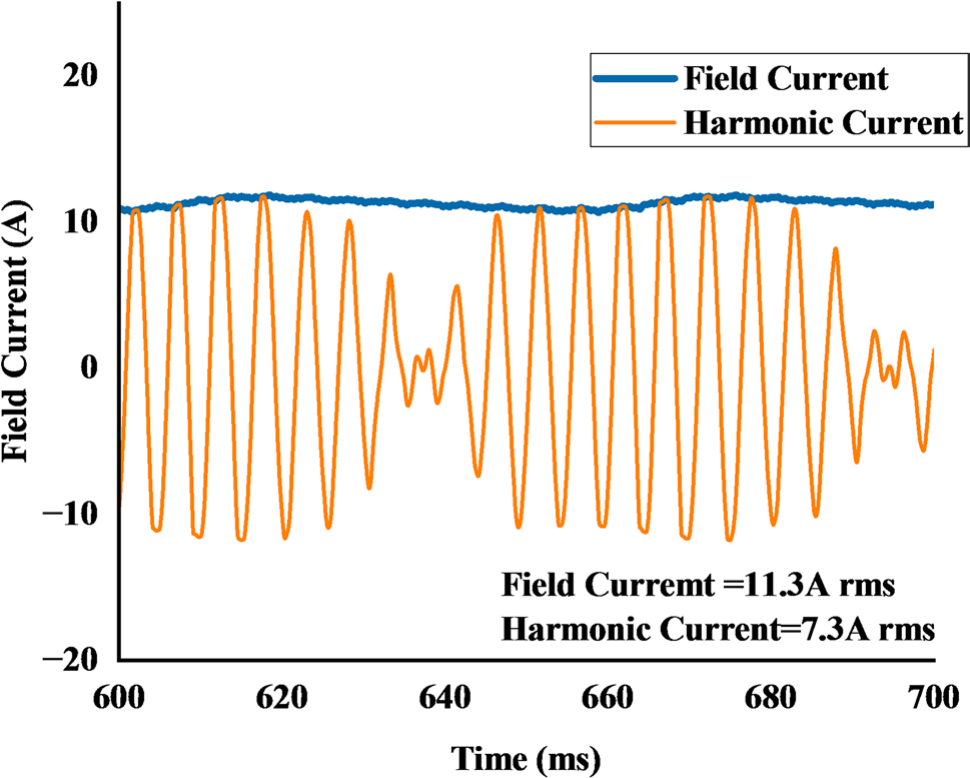
Harmonic and filed current of proposed skewed model.

Figure 21 shows the rotor harmonic and filed current after skewed, the field current is reduced to 11.3 A rms from 11.7 A rms, while harmonic current remained to 7.3 A rms.

**Frequency and core loss** Vernier machines require a high number of rotor poles to generate the magnetic gearing effect (in our case, 44 poles or 22 pole pairs). At the spin-dry speed of 1200 rpm, the electrical frequency required for vernier machines is very high ($\:\mathrm{f}=\frac{1200\mathrm{X}44}{120}=440\mathrm{H}\mathrm{z}$). This high frequency leads to excessive hysteresis and eddy current losses in the core. In contrast, our inner synchronous stator operates with only 2 pole pairs, resulting in a much lower frequency of 40 Hz at the same speed, which drastically reduces core losses.

**Efficiency gap** As shown in Table II, the reference BL-WRVM (an optimized vernier machine) achieves only 70.99% efficiency at high speed. In contrast, our proposed topology switches to the synchronous mode (inner stator), achieving 94.7% efficiency. A nearly 24% improvement in efficiency is a critical advantage for modern household appliances where energy ratings are paramount.

A detailed analysis of the results from simulations is summarized in Table II. Which gives the different performance tradeoffs of the proposed machine topology comparison with Reference BL-WRVM^1^.

In the low speed, the initial Proposed model topology achieved 23.7 Nm average torque more than double from reference model average torque of 9.38 Nm, this high torque comes with tradeoff of higher torque ripple of 73.7% vs. 17%. Further the Proposed Skewed Model successfully reduced the torque ripple to 27% and maintained a torque value of 20 Nm with decreased efficiency from 79.3% to 77%.

In the high-speed dry mode, the proposed non skew machine produced higher average torque of 6.14 Nm compared to the reference model 4.10 Nm with a ripple value of 7% compared to reference model 26%. This comes with highest efficiency of 94.9%, significantly greater than 70.99% of reference model. This efficiency gain is due to decreased copper losses in the inner side of rotor by using magnets and less core losses. Further skewing decreased the ripple to 6.5% from 7% and maintained the torque of 5.9 Nm and an efficiency of 94.7% (Table 2).

**Table 2:** Comparison of results.

Parameters	Unit	Reference BL-WRVM^1^		Proposed		Proposed skewed model	
		Wash mode	Dry mode	Wash mode	Dry mode	Wash mode	Dry mode
Average torque	Nm	9.38	4.10	23.7	6.14	20	5.9
Torque ripple	%	17.00	26.00	73.7	7	27	6.5
Input power	W	653	724	1638	812.9	1423.6	782.7
Output power	W	514	514	1300	771.5	1097.5	741.4
Total core loss	W	87.13	144.23	111	20	110	20
Total copper loss	W	52.55	65.77	226.5	11.82	215	11.82
Efficiency	%	78.71	70.99	79.3	94.9	77	94.7

**Fig. 22:**
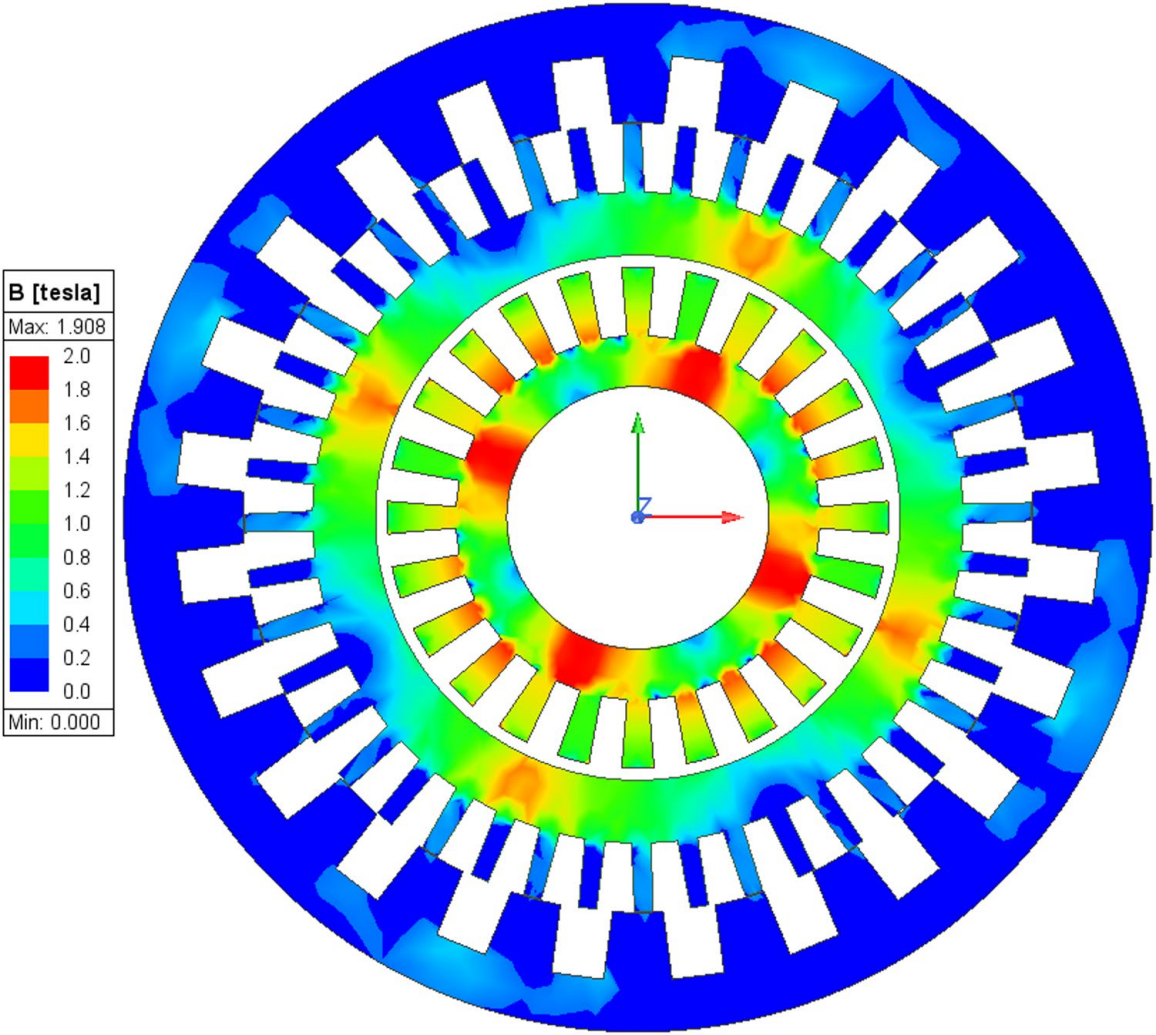
Magnetic flux density (B) of core distribution under loaded conditions in high speed (spin-dry) mode.

**Fig. 23:**
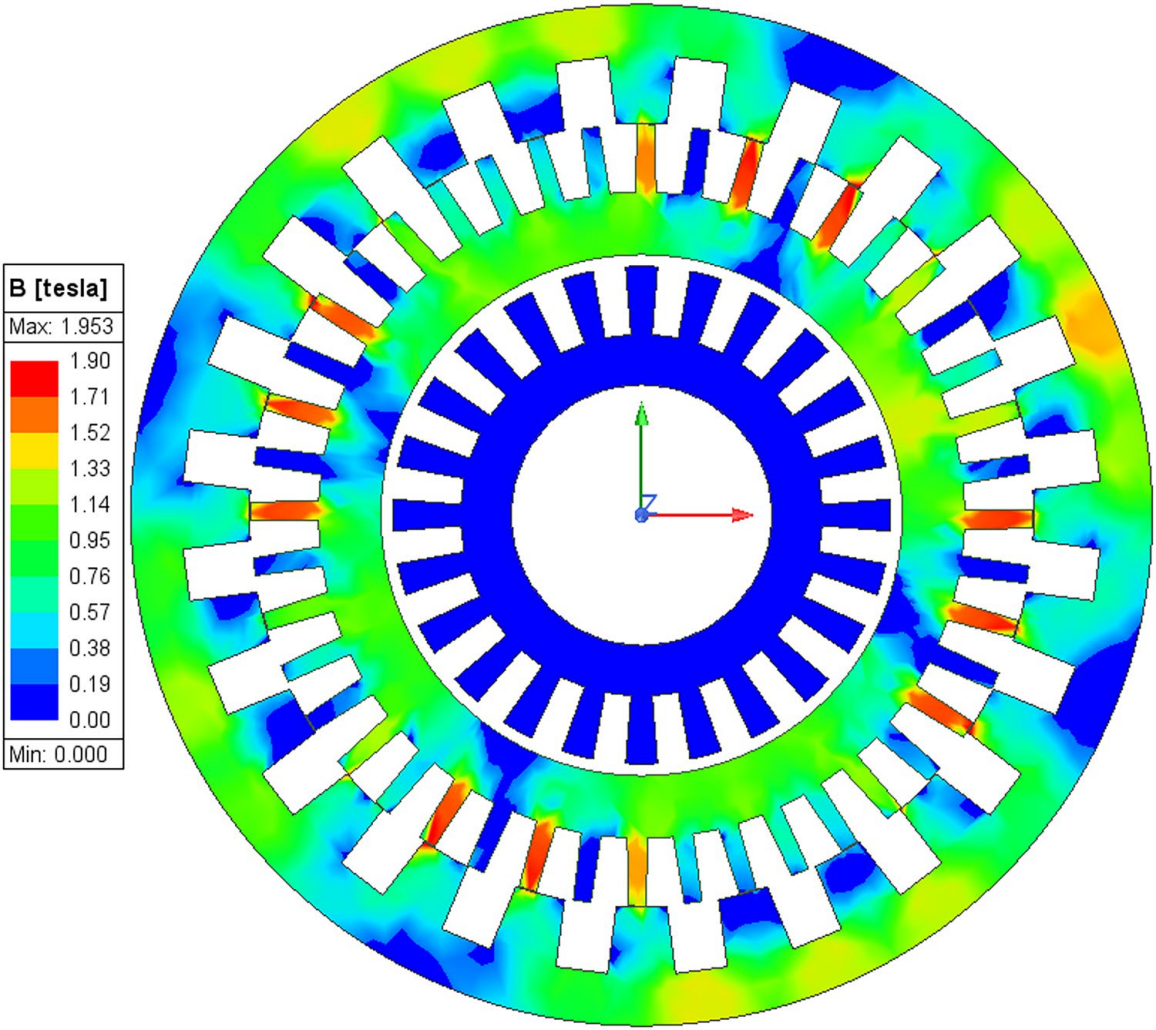
Magnetic flux density (B) of core distribution under loaded conditions in low-speed (wash) mode.

Further an electromagnetic analysis is conducted to show the magnetic flux density in the machine; The flux density plot for high-speed mode at 1200 rpm and for low speed at 524 rpm under load conditions are shown in Figs. 22 and 23 respectively. In this analysis, for synchronous PM mode the magnetic flux is concentrated in the inner stator and the inner PMs of the rotor. From the simulation it shows that the flux density reaches a maximum value of approximately 1.908 T, primarily in the inner stator. Further in brushless vernier mode, the magnetic flux is concentrated in the outer stator and the 44 pole outer rotor teeth. where magnetic flux value reaches approximately 1.97T in outer teeth.

## Conclusion

The study presents a DMDS-HRSVM that combines hybrid excitation, brushless operation, and vernier principles to achieve improved electromagnetic performance for variable speed drive applications. The proposed configuration successfully achieves dual-mode functionality, providing high torque 20 Nm at low speed through sub-harmonic brushless excitation.

While the vernier mode exhibited higher torque ripple (73.7%), further rotor skewing reduced it to 27%, future work on pole-arc optimization, and PM shaping can further smooth torque output.

The proposed dual-stator topology introduces increased mechanical complexity compared to a standard single-stator machine. However, we contend that these mechanical trade-offs are fully justified by the electromagnetic advantages specific to the washing machine application cycle:

- **Decoupled Optimization**: A single-stator machine forces a compromise between low-speed torque and high-speed efficiency. Our dual-stator design eliminates this compromise, allowing the machine to achieve **94.7% efficiency** at high speeds (where Vernier machines typically fail due to core losses).
- **Reduced Magnet Cost**: By using a hybrid rotor that only requires PMs for the inner interface, we significantly reduce the volume of rare-earth magnets required compared to a similarly rated high-torque PMSM.
- **Reliability**: The design successfully eliminates brushes and slip rings, solving the maintenance issues of conventional Wound Rotor Synchronous Machines (WRSMs) while maintaining the flux-weakening control benefits of a wound field.

Overall, the DMDS-HRSVM represents a major advancement in electric-machine engineering by unifying vernier and synchronous technologies in a single magnet-light platform offering high efficiency, reduced maintenance, and broad applicability for modern washing-machine and other variable-speed domestic or traction systems.

## Data Availability

The datasets used and/or analyzed during the current study are available from the corresponding author on reasonable request.
